# In Vitro Toxicity of TiO_2_:SiO_2_ Nanocomposites with Different Photocatalytic Properties

**DOI:** 10.3390/nano9071041

**Published:** 2019-07-21

**Authors:** Rossella Bengalli, Simona Ortelli, Magda Blosi, Anna Costa, Paride Mantecca, Luisa Fiandra

**Affiliations:** 1 POLARIS Research Centre, Department of Earth and Environmental Sciences, University of Milano—Bicocca, Piazza della Scienza 1, 20126 Milano, MI, Italy; 2 Institute of Science and Technology for Ceramics (CNR-ISTEC), National Research Council of Italy, Via Granarolo 64, 48018 Faenza, RA, Italy

**Keywords:** nanoparticles, safe(r)-by-design, in vitro toxicity

## Abstract

The enormous technological relevance of titanium dioxide (TiO_2_) nanoparticles (NPs) and the consequent concerns regarding potentially hazardous effects that exposure during production, use, and disposal can generate, encourage material scientists to develop and validate intrinsically safe design solution (safe-by-design). Under this perspective, the encapsulation in a silica dioxide (SiO_2_) matrix could be an effective strategy to improve TiO_2_ NPs safety, preserving photocatalytic and antibacterial properties. In this work, A549 cells were used to investigate the toxic effects of silica-encapsulated TiO_2_ having different ratios of TiO_2_ and SiO_2_ (1:1, 1:3, and 3:1). NPs were characterized by electron microscopy and dynamic light scattering, and cell viability, oxidative stress, morphological changes, and cell cycle alteration were evaluated. Resulting data demonstrated that NPs with lower content of SiO_2_ are able to induce cytotoxic effects, triggered by oxidative stress and resulting in cell necrosis and cell cycle alteration. The physicochemical properties of NPs are responsible for their toxicity. Particles with small size and high stability interact with pulmonary cells more effectively, and the different ratio among silica and titania plays a crucial role in the induced cytotoxicity. These results strengthen the need to take into account a safe(r)-by-design approach in the development of new nanomaterials for research and manufacturing.

## 1. Introduction

Nanotechnology is growing tremendously fast, and it has led to the creation of a new class of materials, called nanomaterials (NMs), which are nowadays present in everyday life goods. In particular, metal oxide nanoparticles (NPs), thanks to their several properties (antibacterial, photocatalytic, etc.) [1,2] are gaining great attention. Nevertheless, there is a growing concern about the safety of these NMs, due to the increasing release in the market and their consequent intentional and unintentional emission into the environment. In this perspective, to guarantee a sustainable development of these new technologies, the environmental and health safety issues should be addressed in parallel.

Titanium dioxide (TiO_2_) is largely used as a white pigment in the production of several manufacturings (paint, ceramics, textiles, dental care products, etc.) thanks to its ability to strongly adsorb ultraviolet (UV) lights. Under UV light irradiation, the physical properties of TiO_2_ change, promoting the decomposition of organic and inorganic compounds. For these properties, TiO_2_ is highly employed as a photocatalytic agent in a variety of applications, such as pollution remediation in air and water, sterilization, and production of self-cleaning surfaces. Photocatalyzed TiO_2_ nanoparticles (NPs) have been thoroughly examined given their potential application in cancer therapies and their ability in eradicating cancer cells depends on particle concentrations, cell types, and surface chemistry [3]. Recently, TiO_2_ NPs have been identified as efficient sensitizers for photodynamic and sonodynamic cancer therapy, especially upon functionalization with antibodies aimed to optimize the selective distribution to target cells [4].

TiO_2_ nanoparticles (NPs), which are also commonly used in food coloring and packaging, cosmetic and oral care products have recently been investigated as efficient antimicrobial agents. TiO_2_ NPs have long been known as efficient antimicrobial agents [5]. The photocatalytic properties of TiO_2_ NPs depend on its crystalline structure, with the anatase crystalline structure having the highest photocatalytic activity [6,7], and on particle size and specific surface area (the smaller the particle size, the greater the number of active surface sites, and the surface charge-carrier-transfer rate increases in photocatalysis). It has been previously demonstrated that TiO_2_ nanoparticles’ complexation with silica (SiO_2_) increases TiO_2_ photocatalytic activity on self-cleaning textiles, proportionally related to SiO_2_ content. The higher photocatalytic effect has been ascribed to an increase of titanium surface acidity that, in turn, affected its hydrophilicity [8].

The issue of TiO_2_ toxicity is a crucial aspect to be considered for its application as a photocatalytic agent in self-cleaning surfaces. Even if over the last decades the research activity on the toxicity of TiO_2_ NPs has been strongly improved, questions about the safe use of these nanoparticles are still open. As photocatalytic activation of TiO_2_ induces a greater generation of reactive oxygen species (ROS), so it is likely that this also improves its toxicological potential. Some reports from the literature have shown that TiO_2_ is toxic in the presence of UV irradiation due to ROS generation [9]. UV irradiation of TiO_2_ suspensions can also change the state of agglomeration of the nanoparticles affecting their photocatalytic activities [10] with an indirect impact on cell viability. Several in vitro studies evidenced that a certain toxicity and genotoxicity is associated with the exposure to TiO_2_ NPs even in the absence of photoactivation. Oxidative stress is the main toxicity mechanism for non-photoactivated NPs also, with alteration of mitochondrial functionality and induction of DNA damage in different human and non-human cell lines [11,12,13].

The surface functionalization of TiO_2_ NPs with SiO_2_, resulting in an increase of hydrophilicity, not only was demonstrated to be able to improve their photocatalytic potential on textile surfaces [8] but also to affect their biocompatibility. TiO_2_ NPs encapsulation in a SiO_2_ matrix was demonstrated to be an effective solution to control the redox reactivity of TiO_2_ NPs by reducing ROS production [14]. Nevertheless, the toxic effects on live cells, as a function of encapsulating SiO_2_ content, have limited investigations. NPs photocatalyst performance is related to the ratio TiO_2_:SiO_2_ in the nanocomposites, which can also be responsible for different cellular consequences upon direct exposure to these new nanomaterials (NMs).

Recently, studies and development of hybrid and core@shell NPs, nanocomposites, and layer-by-layer NPs have arisen [15,16,17,18]. Since these new NMs have different properties from their single NPs constituents, concern and research about their safety are increasing in parallel, pointing out the importance of investigating NPs toxicity in a perspective of sustainable nanotechnology. This study aims to compare the different biological effects induced by silica-encapsulated TiO_2_ [19], comparing different SiO_2_ content (TiO_2_/SiO_2_ ratio). The effects of the different nanocomposites were tested against the human lung epithelial cells A549, that have been demonstrated as being a good target for the safety assessment of titania NPs [13,20]. Furthermore, A549 cells, derived from a human adenocarcinoma of the lung, are the most often used cell line for toxicity testing. The cells show properties such as surfactant production and transport=like AT-II cells in vivo, secrete cytokines, and perform phase I and phase II xenobiotic biotransformation similar to lung tissue [21,22]. Furthermore, it is important to select the cell line which best represents the intended exposure route, and in this perspective, A549 cells are used for the screening of nanoparticles in inhalation toxicology. Finally, in the context of the NANoREG framework for the safety assessment of NMs [23], A549 cells were used as the main model common for several biological endpoints, making this cell line a promising candidate as a cellular model for in vitro testing of NMs at the regulatory level.

The obtained results are of great importance in the selection of the best SiO_2_-modified TiO_2_ photocatalysts, matching safe-by-design requirements.

## 2. Materials and Methods

### 2.1. Materials

The following commercial materials were used to prepare the TiO_2_:SiO_2_ samples: TiO_2_ colloidal nanosuspension (nanosol) containing 6 wt % titania (Colorobbia Italia SpA, Sovigliana Vinci, Italy) and SiO_2_ colloidal nanosol, Ludox HS-40^®^ containing 40 wt % silica (Grace Davison, Columbia, MD, USA). Dowex^®^ 66 anionic exchange resin was purchased from Sigma-Aldrich (Saint Louis, MO, USA).

### 2.2. TiO_2_:SiO_2_ Nanosuspensions Preparation

The commercial TiO_2_ and SiO_2_ nanosols, after appropriate dilution with distilled water, were mixed in well-defined ratios and ball milled for 24 h (zirconia balls with 5 mm diameter). Weight ratios of 1:1, 1:3, and 3:1 were investigated for TiO_2_:SiO_2_ NPs. The TiO_2_:SiO_2_ samples were mixed at opposite surface charge conditions to promote colloidal heterocoagulation [24]. The obtained samples were basified by anionic exchange resin until a pH of 5 to be more compatible with biological targets. The nanocomposites obtained by the colloidal approach were named TiO_2_:SiO_2_ 1:1, TiO_2_:SiO_2_ 1:3, and TiO_2_:SiO_2_ 3:1 and characterized by a TiO_2_ content equal to 0.75 wt %.

### 2.3. TiO_2_:SiO_2_ Nanoparticles Characterization

The TiO_2_:SiO_2_ nanosols underwent morphological analysis using the FEI Titan transmission electron microscope (TEM) operating at an acceleration voltage of 300 kV (FEI Company, Hillsboro, OR, USA). One drop of the nanoparticle suspension diluted in deionized water (75 μg/mL) was deposited on a film-coated copper grid and characterized.

To perform the identification of crystalline phases, TiO_2_:SiO_2_ 1:1, TiO_2_:SiO_2_ 1:3, and TiO_2_:SiO_2_ 3:1 samples were dried at T° = 100 °C for 3 h in an oven. The obtained powders were characterized by X-ray diffraction (XRD) using a Bragg–Brentano diffractometer (Bruker D8 Advance, Karlsruhe, Germany) operating in a θ/2θ configuration, with an X’Celeretor detector LynkEye (10°–80° 2θ range, 0.02 step size, 0.5 s per step).

To investigate the colloidal behavior of TiO_2_:SiO_2_ NPs in water, hydrodynamic diameter (z-average) and zeta potential (ζ–potential) were measured by dynamic light scattering (DLS) and electrophoretic light scattering (ELS) measurements, respectively, using Zetasizer Nanoseries apparatus (Malvern Instruments, Malvern, UK).

### 2.4. Cells Culture Maintenance and Treatments

The A549 cell line (ATCC^®^ CCL-185™) was routinely maintained as previously reported in Gualtieri and colleagues [25]. Briefly, cells were maintained in OptiMEM medium (Gibco, Life Technologies) supplemented with fetal bovine serum (Gibco, Life Technologies, Monza, Italy) 10% and Pen/Strep 1% (Euroclone, Pero, Italy) at 37 °C, 5% CO_2_.

For the treatments with the different TiO_2_:SiO_2_ NPs, cells were seeded in 6 MW (Corning) at the density of 1.5*10^5^ cells/well and incubated until they attached and reached to the confluence of 80% (about 24 h). Cells were treated with NPs for 24 h in all experiments, except for cytofluorimetric analysis (ROS and uptake) in which they were exposed for 180 min (3 h). Each experiment included negative control (untreated cells) and positive control (1 mM H_2_O_2_ for ROS detection). For the cell viability experiments, cells were treated with different doses of TiO_2_ (7.5, 75, and 750 μg TiO_2_/mL), while for the other experiment, a dose of 75 µg TiO_2_/mL was used.

### 2.5. Cell Viability

The cytotoxicity of TiO_2_:SiO_2_ was evaluated through two different analysis: by the 3-(4,5-dimethylthiazol-2-yl)-2,5-diphenyltetrazolium bromide (MTT) test and Hoechst 33342/propidium iodide (H/PI) staining.

MTT (Sigma Aldrich, Milano, Italy) assay was performed according to previous works [26]. Briefly, after 24 h of exposure to NPs, supernatants were collected, centrifuged at 1200 rpm for 6 min, and stored at −80 °C. Cells were rinsed with phosphate buffered saline (PBS), and MTT solution was added to the medium (final concentration 0.3 mg/mL) for 2 h. After the formazan crystal, which are the MTT reduction products, were dissolved in 1 mL Dimethyl sulfoxide (DMSO, Sigma Aldrich), absorbance was analyzed at 570 nm by a multi-plate reader (Infinite 200 Pro, TECAN, Männedorf, Switzerland).

The H/PI nuclear staining allowed to count the number of viable, necrotic, apoptotic, and mitotic A549 cells after NPs exposure. Cells were detached by trypsinization and then re-suspended in a complete medium; 10 μL of 1:1 solution of Hoechst 33342/PI (Sigma Aldrich) was added to cell suspensions and stored in the dark for 15 to 30 min at room temperature. Cells were then centrifuged for 6 min at 1200 rpm, and re-suspended in 20 μL of FBS. Three drops (about 4 μL) of cell suspensions were smeared on a coverslip and observed under a fluorescence microscope (Zeiss-Axioplan, Carl Zeiss Microscopy GmbH, Jena, Germany) with a UV filter (365 nm). At least 300 cells per sample were scored according to nuclei staining and plasma membrane integrity as viable normal cells (H positive and PI negative, without special nuclear characteristic and an intact plasma membrane), necrotic cells (non-apoptotic and PI positive), apoptotic cells (bright H or PI positive stained with condensed or fragmented nuclei), mitotic cells (H positive with chromosome condensation).

### 2.6. Cytological Observation

For morphological analysis, cells were seeded on a cover slide at a concentration of 1.5*10^5^ cells/well, cultured for 24 h and then exposed to NPs for further 24 h. At the end of the treatment, cells were processed for hematoxylin-eosin (HE) staining, as previously described in [27]. The slides were observed on an optical microscope (Zeiss-Axioplan, Carl Zeiss Microscopy GmbH, Jena, Germany), and pictures were acquired using an AxioCam MRc5 digital camera and processed using AxioVision Real 4.8 software (Carl Zeiss Solutions, Jena, Germany).

Cytoskeleton organization was instead analyzed by staining actin microfilaments with rhodamin phalloidine (1:40 dilution, Cytoskeleton Inc., Denver, CO, USA). Cells, seeded on a cover slide, after exposure to 75 µg/mL of NPs, were fixed in 4% paraformaldehyde for 20 min, washed and permeabilized with 0.1% Triton-100X and then incubated with rhodamin phalloidine for 30 min. After PBS washing, slides were mounted with ProLong™ Gold Antifade Mountant with DAPI (Molecular Probe, Life Technologies, Monza, Italy), dried overnight and then observed under the inverted microscope AxioObserver Z1 Cell Imaging station (Carl-ZEISS Spa, Milano, Italy) and images were acquired by an MRc5 digital camera and elaborated with the dedicated software ZEN 2.3 Blue edition.

### 2.7. Cytofluorimetric Analysis

Cytofluorimetric analyses were performed: (i) to evaluate TiO_2_:SiO_2_ NPs capability to induce ROS formation, (ii) to investigate the interaction between different NPs with A549 cells by the side scatter analysis, and (iii) to analyze cell cycle alterations.

For ROS detection, A549 cells were pre-incubated for 20 min with the probe Carboxy-2′,7′-Dichlorofluorescein Diacetate (carboxy-DCFDA, 5 μM, Life Technologies) and incubated with NPs for 180 min. After incubation, cells were detached, centrifuged at 1200 rpm for 6 min, re-suspended in 500 μL of PBS and analyzed in the FITC channel by flow cytometry (CytoFLEX, Beckman Coulter GmbH, Krefeld, Germany) with the software CytoExpert. The signal from unloaded samples (cells without the probe carboxy-DCFDA) was evaluated as reference to assess cells and NPs’ autofluorescence. These values were then subtracted from the values to DCFDA stained samples.

To investigate the effects on cell cycle, untreated and cells treated for 24 h were harvested, fixed in ethanol 90% and stored at −20 °C until analysis. Cells were then centrifuged at 1600 rpm for 6 min, ethanol was discharged, and cells suspended in PBS containing RNAsi-DNasi free (10 µL) for 30 min at 37 °C. Then 5 µL of Propidium Iodide (PI) was added for 7 min and finally, cells analyzed by the Cytoflex in the ECD-channel (λEcc 488 nm, λEm 610/20 nm). The percentage of cells in the different cell cycle phase (sugG0, G1, S, and G2/M) was displayed. The side scatter (SSC) was also analyzed to assess cell–NP interaction.

Furthermore, the capability of the different NPs to induce autophagy was assessed through the analysis of LC3B II expression by cytofluorimeter. Cells, after 24 h of exposure to 75 µg/mL of NPs, were harvested, fixed with paraformaldehyde 4% for 15 min, permeabilized with methanol 90%, and then stained with the antibody rabbit anti-human LC3BII (1:400 dilution, Cell Signaling). After 1 h of incubation in the appropriate buffer (0.5% bovine serum albumin in PBS 1X), cells were washed twice in buffer and then incubated for 30 min with secondary antibody AlexaFluor anti-rabbit 488 (1:200 dilution, Life Technologies). After washing, cells were resuspended in PBS and read by the CytoFLEX in the FITC channel.

### 2.8. Statistical Analysis

The data represent the mean ± standard error of the mean (SEM) of at least three independent experiments. Statistical analyses were performed using Sigma Stat 3.2 software, using unpaired *t*-test or one-way ANOVA and Bonferroni’s post hoc analysis. Values of *p* < 0.05 were considered statistically significant.

## 3. Results

### 3.1. TiO_2_:SiO_2_ Nanocomposites Characterization

#### 3.1.1. Transmission Electron Microscopy (TEM)

**Figure 1:**
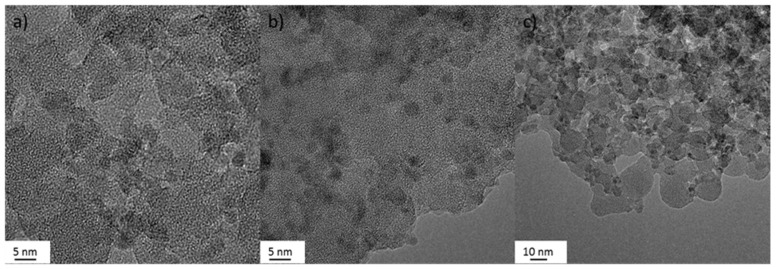
TEM images of TiO_2_:SiO_2_ sample: (**a**) TiO_2_:SiO_2_ 1:1, (**b**) TiO_2_:SiO_2_ 1:3, (**c**) TiO_2_:SiO_2_ 3:1.

**Figure 2:**
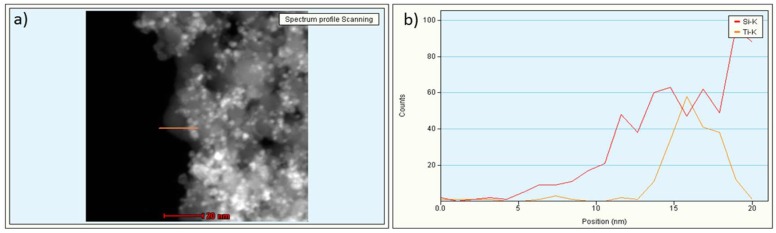
EDX-STEM analysis on the TiO_2_:SiO_2_ 1:1 sample: (**a**) sample area analyzed; (**b**) Si and Ti distribution within the selected area confirm the phase gradient represented in the sketch.

The TEM micrographs (Figure 1) showed aggregated samples with TiO_2_ randomly distributed within a silica matrix. This matrix encapsulation structure is typical of the colloidal heterocoagulation method used to prepare TiO_2_:SiO_2_ samples [19,28]. The energy-dispersive X-ray (EDX) line scan analysis of the elemental distribution, on TiO_2_:SiO_2_ 1:1 sample, confirmed the presence of a matrix encapsulation structure, where the TiO_2_ NPs were surrounded by SiO_2_ NPs (Figure 2).

Data on TiO2 and SiO2 NPs morphology are shown in Supplementary materials (Figure S1).

#### 3.1.2. Colloidal Properties

Data about the hydrodynamic behavior, PdI and ζ-potential values of TiO_2_:SiO_2_ NPs are reported in Table 1. As expected, TiO_2_ (+38 mV) and SiO_2_ (−43 mV) NPs were positively and negatively charged, respectively, at natural pH in water. Their opposite surface charge triggers the heterocoagulation phenomenon, which, by means of electrostatic interactions, promotes the encapsulation of TiO_2_ NPs into the SiO_2_ matrix. The small size, low polydispersity index (PdI) and high values of zeta-potential demonstrated the good colloidal stability of both starting NPs suspensions. The increase of hydrodynamic diameter as a function of the TiO_2_:SiO_2_ ratio is caused by both the steric hindrance of SiO_2_ heterocoagulated on the TiO_2_ surface and the consequent electrostatic destabilization due to the progressive neutralization of the TiO_2_ surface charge with the increase of SiO_2_ content [29,30]. The hydrodynamic diameters showed that TiO_2_:SiO_2_ NPs 1:1 and 3:1 have similar behavior, with average values of 126 and 148 nm, respectively. On the other hand, the hydrodynamic diameter of the sample TiO_2_:SiO_2_ 1:3 was much larger than the other NPs (1490 nm) evidencing its tendency to aggregate in an aqueous medium. Moreover, TiO_2_:SiO_2_ 1:3 also showed the highest PdI, consistent with a very broad size distribution. Such a different behavior among the samples was well expressed by the zeta potential values. A positively charged surface of the NPs was measured for all the heterocoagulated suspensions. The zeta potentials of TiO_2_, TiO_2_:SiO_2_ 1:1 and TiO_2_:SiO_2_ 3:1, higher than +35 mV, pointed out that these suspensions are stable. On the contrary TiO_2_:SiO_2_ 1:3 exhibited a low ζ-potential value (<+10 mV), meaning that the electrostatic repulsion barrier cannot prevent flocculation and coagulation phenomena.

#### 3.1.3. XRD-Analysis

**Figure 3:**
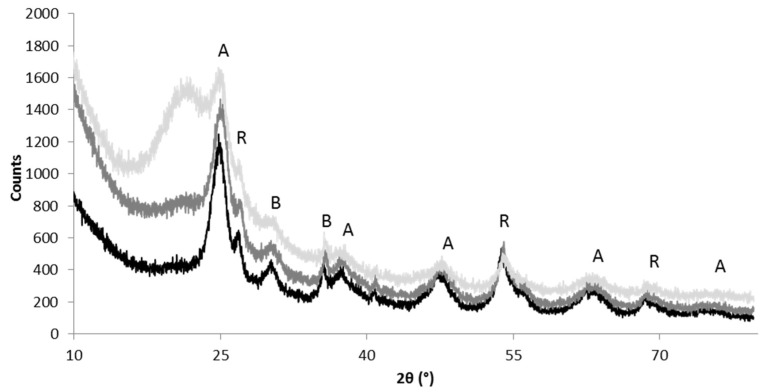
X-ray diffractograms of TiO_2_SiO_2_ 3:1 (black), TiO_2_SiO_2_ 1:1 (medium gray) and TiO_2_SiO_2_ 1:3 (light gray). (A = anatase; B = brookite; R = rutile).

Figure 3 shows the XRD patterns for TiO_2_SiO_2_ 3:1, TiO_2_SiO_2_ 1:1 and TiO_2_SiO_2_ 1:3 powder samples. The samples show a broad band centered at 2θ = 22.0, the characteristic peak for amorphous SiO_2_ (JCPDS 29–0085) with an intensity proportional to the amount of silica added. Anatase (JCPDS 21–1272) is the predominant crystalline phase of TiO_2_ component, then there are small amounts of brookite (JCPDS 29–1360) and rutile (JCPDS 65–0190). In general, broad peaks are present, they are typical of nano-sized crystallites.

### 3.2. Cytotoxicity

#### 3.2.1. Cell Viability: MTT Test and H/PI

**Figure 4:**
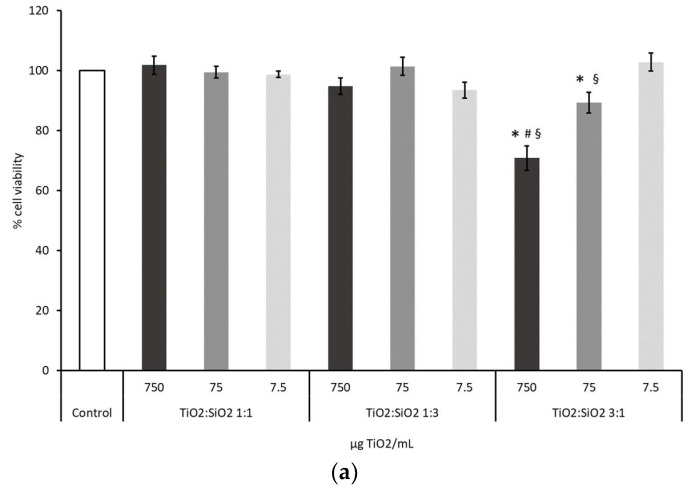

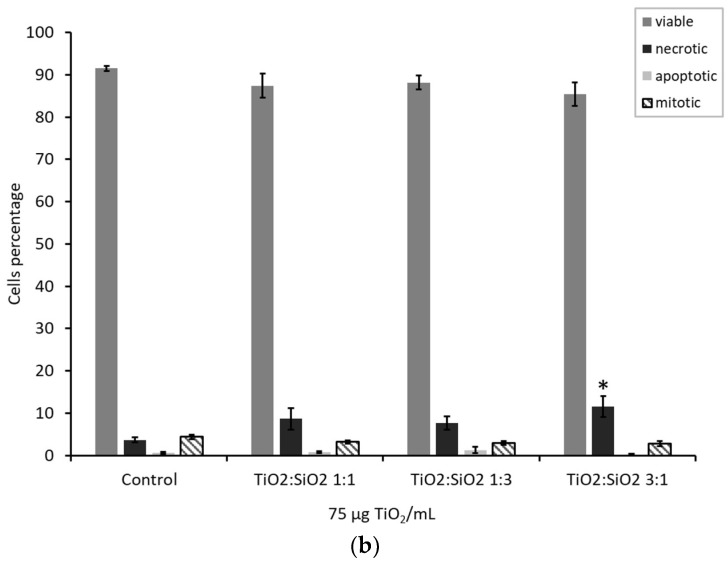
(**a**) Cell viability was assessed by MTT test after 24 h of exposure to increasing doses of different TiO_2_:SiO_2_ NPs. The white histogram represents control sample, dark grey bars represent dose 750 µg TiO_2_/mL, grey bar dose 75 µg TiO_2_/mL, and light grey bars dose 7.5 µg TiO_2_/mL. Data show the mean ± SE of at least three independent experiments. * Statistically significant with respect to control, *p* < 0.001; ^#^ Statistically different with respect to TiO_2_:SiO_2_ 1:1 at dose 750 µg TiO_2_/mL, *p* < 0.001; ^§^ Statistically different with respect to TiO_2_:SiO_2_ 1:3 at dose 750 µg TiO_2_/mL, *p* = 0.006. One-way ANOVA + Bonferroni’s post hoc test. (**b**) Hoechst 33342/propidium iodide (H/PI) staining of A549 treated for 24 h with different TiO_2_:SiO_2_ NPs (75 µg TiO_2_/mL). The histograms represent the percentage of viable (grey bars), necrotic (black bars), apoptotic (light grey bars), and mitotic (dashed bars) cells. Data show the mean ± SE of at least three independent experiments. * Statistically significant with respect to control according to one-way ANOVA + Bonferroni’s post hoc test; *p* < 0.05.

Cells viability was assessed to evaluate the toxic potential of TiO_2_ based nanocomposites. Data from MTT test showed that particles TiO_2_:SiO_2_ 3:1, with a lower amount of SiO_2_ vs. TiO_2_, have affected the viability of the cells at the doses 750 and 75 µg/mL with a mortality of the 30% and 10%, respectively (Figure 4a). Cell viability data obtained from the exposure to TiO_2_ and SiO_2_ NPs alone are reported in Supplementary Materials (Figure S2). To investigate the type of cell death induced by these NPs, H/PI staining has been performed at the dose 75 µg TiO_2_/mL, which is the dose used for the other endpoints. Furthermore, this effective concentration falls in the range (from 1 µg/mL until 200 µg/mL) recommended for the in vitro testing of NMs aiming to investigate biological responses and in mechanistic studies [31,32]. Results from this test showed that TiO_2_:SiO_2_ 3:1 induced a reduction of cell viability, as demonstrated by the increased number of necrotic cells (Figure 4b). Results on TiO_2_ NPs alone are reported in the Supplementary Materials (Figure S3a).

Data were also confirmed by the Annexin V/PI test, which evidenced that the exposure to TiO_2_:SiO_2_ 3:1, as well as TiO_2_ NPs alone, increased the percentage of early necrotic cells (Annexin V+/PI+). Thanks to the Annexin V test, a slight increase of apoptotic (Annexin V+) cells, after exposure to TiO_2_:SiO_2_ 1:1, 3:1, and TiO_2_ NPs alone, was appreciated (Supplementary Materials S3b,c).

#### 3.2.2. Oxidative stress: Reactive Oxygen Species (ROS) Formation

**Figure 5:**
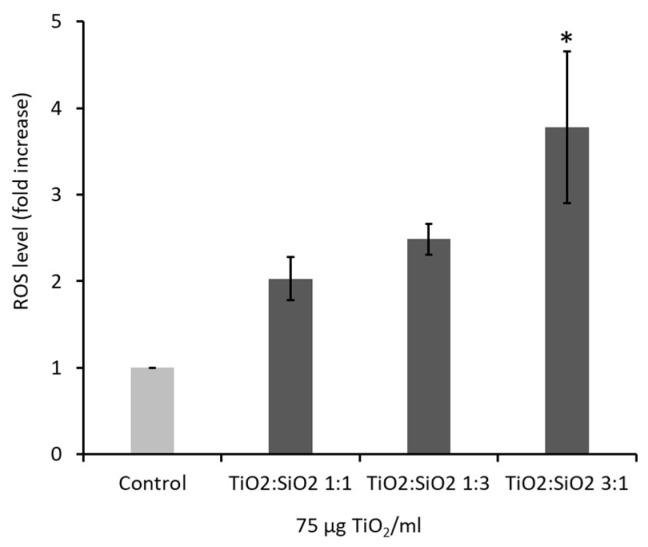
Oxidative stress was evaluated by detecting ROS in A549 after 180 min of exposure to TiO_2_:SiO_2_ NPs (75 µg TiO_2_/mL) by using the fluorescent probe DCFDA. The histograms represent the mean ± SE of at least three independent experiments. * Statistically significant with respect to control; One-way ANOVA + Bonferroni’s post hoc test; *p* < 0.05.

The induction of oxidative stress was evaluated by measuring the intracellular levels of ROS at early time points (180 min). Data showed that only TiO_2_:SiO_2_ 3:1 NPs at the dose of 75 µg/mL was able to induce a significant increase of ROS levels (3.5-fold) with respect to control (Figure 5). Data on TiO_2_ and SiO_2_ NPs alone are reported in the Supplementary Materials (Figure S4).

#### 3.2.3. Cell Cycle Analysis

**Figure 6:**
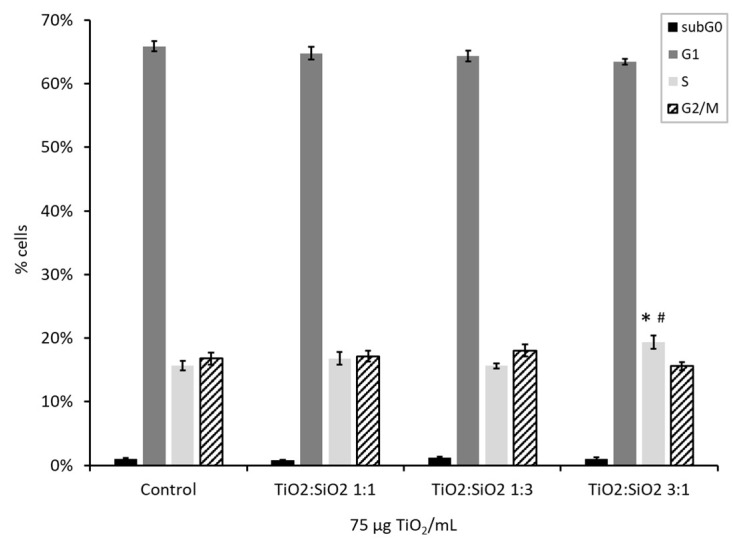
Different phases of the cell cycle (subG0, G1, S, and G2/M) were evaluated through Propidium Iodide content by cytofluorimetric analysis. The histograms represent the mean ± SE of at least three independent experiments. * Statistically significant with respect to control sample; ^#^ Statistically significant with respect to the sample TiO_2_:SiO_2_ 1:3. Unpaired *t*-test, *p* < 0.05.

The analysis of the cell cycle allows us to understand the potential of NPs to induce DNA damage. Data showed that TiO_2_:SiO_2_ 3:1 NPs at the dose of 75 µg/mL induced a slight but significant arrest of the cell cycle in the S-phase (Figure 6). In fact, in control sample, 15.6% of cells were in the S-phase, while this percentage of cells is increased to 19.3% when treated with TiO_2_:SiO_2_ 3:1 NPs.

#### 3.2.4. NPs Induced Autophagy: LC3B II Expression

**Figure 7:**
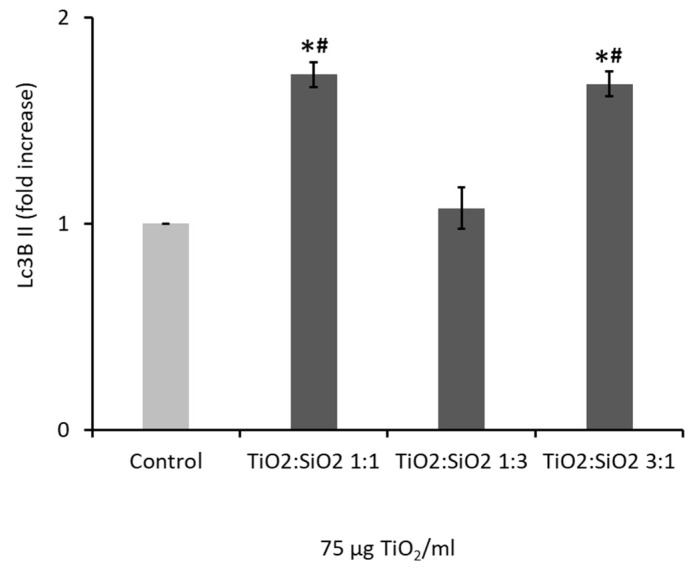
Autophagy was investigated through the analysis of the expression of LC3B II protein by cytofluorimeter. Cells were stained with LC3B II antibody after 24 h exposure to TiO_2_:SiO_2_ NPs (75 µg TiO_2_/mL). The histograms represent the fold change of LC3B II expression over control, and they are the mean ± SE of at least three independent experiments. * Statistically significant with respect to control; ^#^ Statistically different from sample TiO_2_:SiO_2_ 1:3; *t*-test + Bonferroni’s post hoc test; *p* < 0.05.

The analysis of the LC3BII protein allows us to understand the potential of NPs to induce cell autophagy. Data showed that TiO_2_:SiO_2_ 1:1 and 3:1 NPs at the dose of 75 µg/mL induced a significant increase of LC3BII expression, while 1:3 NPs values are similar to the control one (Figure 7). Data on TiO_2_ and SiO_2_ NPs alone are reported in the Supplementary Materials (Figure S5).

### 3.3. Cell Morphology and NPs Interaction

#### 3.3.1. Morphological Analysis

**Figure 8:**
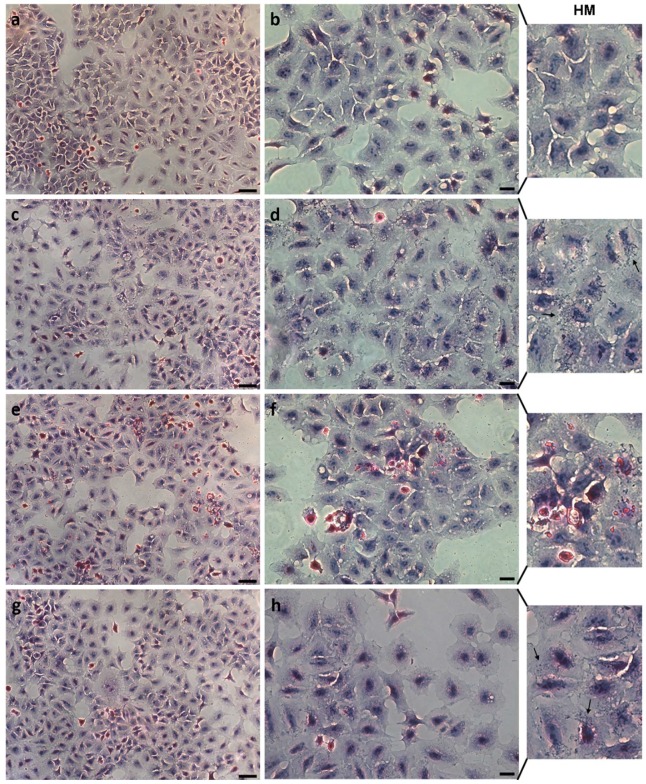
Morphology of cells after exposure to 75 µg TiO_2_/mL of TiO_2_:SiO_2_ NPs 1:1 (**c**,**d**), 1:3 (**e**,**f**), 3:1 (**g**,**h**). Control cells are shown in (**a**,**b**). Cells were fixed and stained with Hematoxylin/Eosin. Scale bars: 50 µm (**a**,**c**,**e**,**g**); 20 µm (b,d,f,h). HM: high magnification of (**b**,**d**,**f**,**h**). Black arrows: nanoparticles interacting with cells.

Data from HE staining showed that the exposure to the different NPs does not induce any significant morphological change (Figure 8). High magnification images (HM) clearly show the interaction of TiO_2_:SiO_2_ NPs 1:1 and 3:1 with A549 cells, while an analog localization of NPs is not detectable in the cells exposed to TiO_2_:SiO_2_ 1:3. On the contrary, high dimension aggregates are visible on these cells. Data on TiO_2_ NPs alone are reported in the Supplementary Materials (Figure S6).

**Figure 9:**
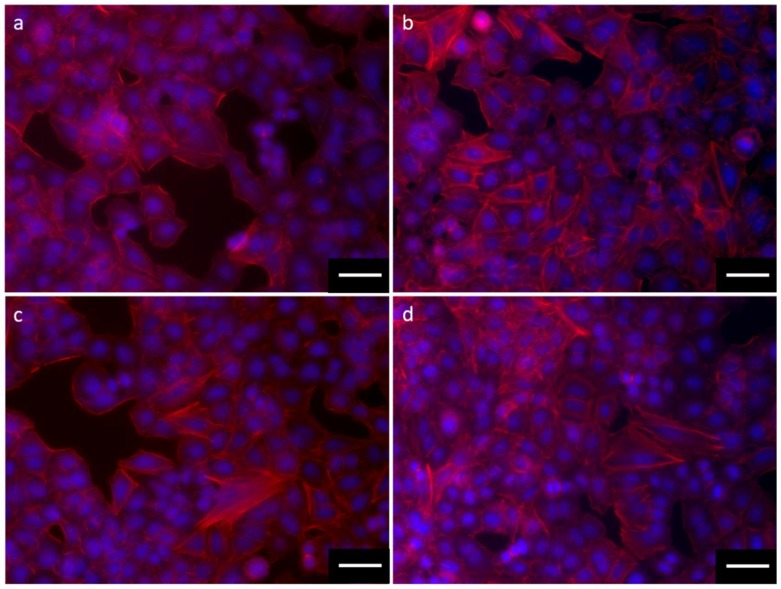
Morphology of cells after exposure to 75 µg TiO_2_/mL of TiO_2_:SiO_2_ NPs 1:1 (**b**), 1:3 (**c**), 3:1 (**d**). Control cells are shown in (**a**). Cells were fixed and stained with DAPI (blue) and Rhodamine Phalloidin (red). Scale bars: 50 µm.

Furthermore, data from rhodamine phalloidin staining showed that the exposure to the different NPs seems to induce actin network morphological change (Figure 9). The interaction of TiO_2_:SiO_2_ NPs 1:1 (Figure 9b) and 3:1 (Figure 9d) with A549 cells, increased cytoskeleton disassembly and the presence of cellular filopodia, while an analog localization of actin is not detectable in the cells exposed to TiO_2_:SiO_2_ 1:3 (Figure 9c). Data on TiO_2_ NPs alone are reported in the Supplementary Materials (Figure S7).

#### 3.3.2. Cell–NP Interactions

**Figure 10:**
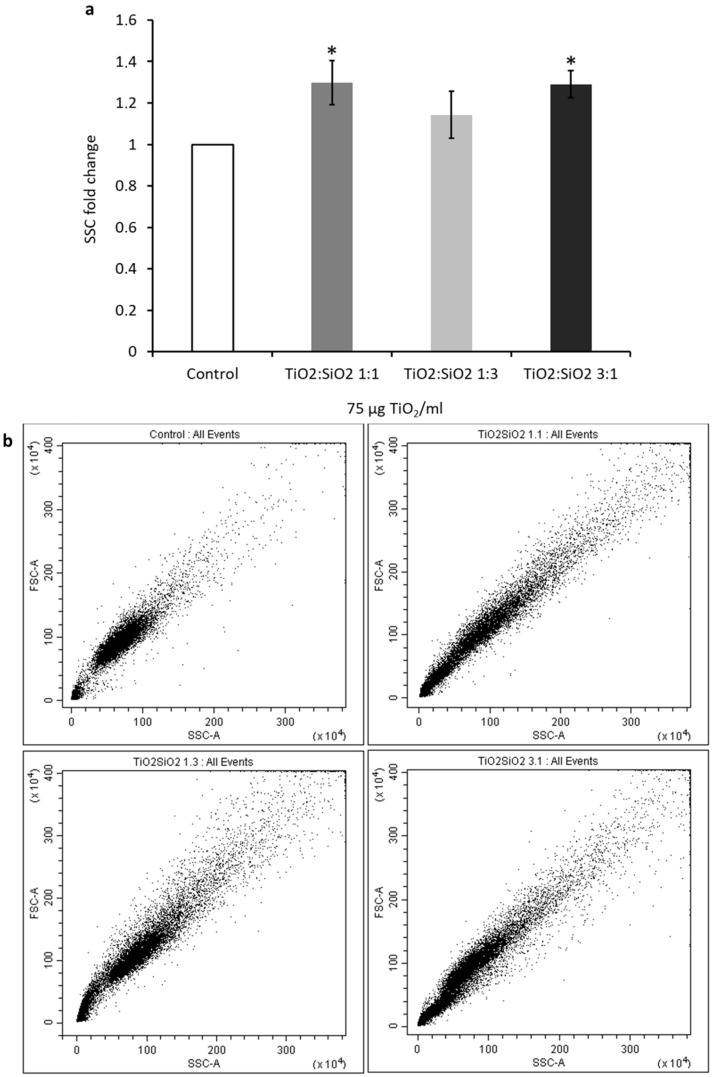
Cell and NP interactions after exposure for 24 h to TiO_2_:SiO_2_ NPs. (**a**) The histograms show the fold change of side scatter (SSC), and data represent the mean ± SE of at least three independent experiments. (**b**) Representative dot-plots of forward scatter (FCS, *y*-axis) and side scatter (SSC, *x*-axis) of untreated cells (Control) and cells exposed for 24 h with TiO_2_:SiO_2_ 1:1 (1.1), 1:3 (1.3), and 3:1 (3.1) NPs. * Statistically significant with respect to control sample; unpaired *t*-test; *p* < 0.05.

The interaction among cells and NPs was investigated by analyzing the side scatter (SSC) of cells through cytofluorimetric analysis. Data showed that there is an increase of 1.3-fold in the SSC with TiO_2_:SiO_2_ 1:1 and 3:1 NPs (Figure 10). This result confirms the higher interaction with A549 cells of TiO_2_:SiO_2_ 1:1 and 3:1 NPs, as suggested by optical microscopy images, in line with the smaller size and more positive surface charge of these nanocomposites with respect to TiO_2_:SiO_2_ 1:3 (Table 1). Data on TiO_2_ NPs alone are reported in the Supplementary Materials (Figure S8).

## 4. Discussion

Crystalline TiO_2_ recently has gained great attention thanks to its special role in a wide range of applications in areas of photovoltaics, photocatalysis, photochromics, and sensors [33]. The employment of TiO_2_ in industry has been a reality for almost 20 years [34], and TiO_2_-based nanomaterials are currently applied in a wide range of manufacturing fields (i.e., personal care, inks, plastics, food products, textiles, and medical devices) [35] and in the development of new anticancer therapies [36,37].

Materials coated with TiO_2_ have long been described for their anti-bacterial properties [38]. Furthermore, TiO_2_ nanotube coated surfaces can enhance osteoblast adhesion and proliferation [39]. Data on murine osteogenic cells demonstrated that the TiO_2_ nanotube arrays can reduce *Staphylococcus epidermidis* colonization and enhance C3H10T1/2 cell adhesion and this dual effect is due to the multiple physical and chemical properties of the TiO_2_ nanotube surface [40]. In 2016, Hashizume et al. demonstrated that after TiO_2_ exposure alteration of the pulmonary inflammatory response may occur and may depend on the surface coating material. Therefore, the pulmonary toxicities of coated TiO_2_ need to be further evaluated, and the physicochemical properties may be useful for predicting the pulmonary risk posed by new nano-TiO_2_ materials [41].

It is likely that the use of TiO_2_ and titania-based nanoparticles as antimicrobial devices is still limited by possible adverse effect on human health, especially upon the skin and pulmonary exposure [42]. Inhalation of engineered NPs, including TiO_2_ NPs, mainly occurs during their production and use in laboratories, industries, and factories, but it is important to consider the whole nanomaterial lifecycle from the perspective of possible releases into the environment. TiO_2_ NPs have been widely investigated for their cytotoxic effects, mainly due to their capability to induce oxidative stress under UV-irradiation [9], but also when not photoactivated [11,12,13]. A recent study has reported how airborne TiO_2_ NPs can induce oxidative stress under outdoor conditions, including UV-irradiation and relative humidity [43], corroborating the crucial issue of TiO_2_ NPs risk assessment, due to the widespread distribution of nanomaterials in the environment [44,45].

In the last decade, different TiO_2_:SiO_2_ nanostructures were designed to enhance TiO_2_ photocatalytic effect [8,46], but TiO_2_ encapsulation in SiO_2_ matrix was also demonstrated effective in reducing ROS production. The protective role of SiO_2_ on TiO_2_-induced oxidative stress was demonstrated by spectroscopic quantification of free radicals production by spin trap molecules [14], suggesting a promising safety outcome in live cells.

In the current study, a comparison of cytotoxicity of TiO_2_:SiO_2_ NPs having different ratios of TiO_2_ and SiO_2_ was performed by analyzing their effects on A549 viability, cell cycle, morphology, and oxidative stress. Cells viability was first assessed after exposure to TiO_2_:SiO_2_ 1:1, TiO_2_:SiO_2_ 1:3, and TiO_2_:SiO_2_ 3:1 with an NP concentration ranging from 7.5 to 750 µg TiO_2_/mL, which is representative of realistic human exposure [47].

A significant impact on cell viability was observed only with TiO_2_:SiO_2_ 3:1 at the higher concentrations and necrosis, and slight apoptosis, seems to be the main pathways of cell death. A549 necrosis induced by exposure to 75 µg TiO_2_/mL TiO_2_:SiO_2_ 3:1 is mediated by increasing reactive oxygen species, according to mechanisms widely described in literature concerning NPs cytotoxicity [48,49].

The comparison with the biological effects induced by the exposure TiO_2_ and SiO_2_ NPs alone is quite controversial, but to have a complete overview of the differences between diverse NMs properties, several endpoints have also been investigated after TiO_2_ and SiO_2_ treatment.

In particular, cytotoxicity was investigated for both SiO_2_ and TiO_2_ NPs alone and data revealed that SiO_2_ NPs are highly toxic even at the intermediate dose of exposure, while TiO_2_ induced cell reduction only at the dose of 750 µg/mL. Compared to cytotoxicity TiO_2_ NPs values, TiO_2_:SiO_2_ 3:1 NPs resulted more toxic. These could be explained by the fact that the nanocomposites are physically different from TiO_2_ and SiO_2_ single NPs nanoparticles. This evidence is confirmed by TEM and DLS results and from previous data from Ortelli et al. [8], in which authors have demonstrated that in an aqueous medium TiO_2_/SiO_2_ samples have a different behavior/reactivity towards water compared to titania and silica alone. The presence of SiO_2_ in the TiO_2_ matrix increased the number of adsorbed molecules without modifying adsorption energy. The addition of SiO_2_ caused an increase in the surface hydrophilicity of titania. Even if the TiO_2_ NPs used in these works are not highly cytotoxic, there are evidences of induced biological effects, such as necrosis, autophagy, ROS induction, and morphological changes (Supplementary Materials). Furthermore, SiO_2_ NPs, which have a very low size (20 nm), are even more toxic of TiO2 NPs alone and TiO_2_:SiO_2_ NPs. Nevertheless, TiO_2_ and SiO_2_ NPs alone are not good photocatalysts, and for these reasons, these NPs were combined to improve TiO_2_ effectiveness in a safe manner.

The lack of any significant effect of colloidal TiO_2_:SiO_2_ 1:3 NPs on the oxidative state of living A549 cells is in line with previous results obtained by Ortelli et al. using a comparable concentration of NPs for a longer exposure time [24]. Moreover, differently from the previous work of Ortelli and colleagues, in the present work, the effect on cells viability, cells cycle, and cell–NP interaction was investigated and nanocomposites with different of TiO_2_:SiO_2_ ratio compared, pointing out that the modification of physicochemical properties, by safe-by-design approach, is crucial in estimating potential in vitro biological responses [50].

Data about the cytotoxic effects of silica-coated-TiO_2_ are in line with in vivo findings on mice, in which these NMs induced pulmonary and sensory irritation after single and repeated exposure and airflow limitation and pulmonary inflammation after repeated exposure [51].

DNA damage is one of the major effects of ROS production [52], and analysis of cell cycle phases is a fast screening for the evaluation of possible alteration at the DNA level after exposure to NPs. Alteration of the cell cycle is, therefore, a marker of genotoxicity. We observed that TiO_2_:SiO_2_ 3:1 was able to induce a slight but significant arrest of the cell cycle in the S-phase. Previous data have reported that TiO_2_ NPs cause arrest in the G2/M-phase [53], even if other evidences have reported a TiO_2_ NPs-induced G1/S-phase arrest due the presence of DNA strand breaks as a consequence of oxidative stress. The arrest in the G1/S and S-phase has also been previously demonstrated in response to other nanoparticles (e.g., ZnO or AuNPs) and with different cell targets [54].

In 2015, Wang et al. demonstrated that TiO_2_ NPs can inhibit A549 cell proliferation, cause DNA damage, and induce apoptosis via a mechanism primarily involving the activation of the intrinsic mitochondrial pathway, suggesting that exposure to TiO_2_ NPs could cause cell injury and be hazardous to health [55]. Furthermore, in vivo experiments showed that exposure in mice to different ratios of SiO_2_:TiO_2_ NPs, induced different effects on DNA damage evidencing that SiO_2_:TiO_2_ composite induced in vivo toxicity, oxidative DNA damage, bargain of the antioxidant enzymes, while SiO_2_:ZrO_2_ composites revealed a lower toxicity in mice compared with that of TiO_2_ [56]. These results confirmed that TiO_2_ NPs can potentially cause adverse effects on organ, tissue, cellular, subcellular, and protein levels due to their unusual physicochemical properties [57].

Another molecular mechanism activated by metal oxide NPs exposure, including titanium dioxide TiO_2_ NPs, is the autophagic pathway associated with toxic effects [38,58,59]. Dysfunction in autophagy could be due to the metal oxide NPs’ ability to increase oxidative stress and cationic damage. Autophagy is an evolutionarily conserved cellular quality control process, and in the case of toxicant and/or metal-induced toxicity, autophagy may act as a survival mechanism by targeting novice components to the lysosomes for degradation [60]. However, excess autophagy may also lead to cell death. Lopes and colleagues [61] showed that the uptake of TiO_2_ NPs leads to a dose-dependent increase in autophagic effect under non-cytotoxic conditions as a cellular response to TiO_2_ NPs and they suggested that simple toxicity data are not enough to understand the full impact of TiO NPs and their effects on cellular pathways or function.

Microtubule-associated protein light-chain protein-3 (LC3) is a key protein in autophagosome formation, and it is converted from its cytosolic form (LC3 I) into an active membrane-bound form (LC3 II) by sequential proteolysis and lipidation during autophagosome assembly [54]. Our data on LC3B II expression confirm that autophagy occurs with TiO_2_:SiO_2_ 1:1, 3:1, and TiO_2_ NPs, confirming that autophagy is one of the main pathways involved in the response to metal oxide NPs.

Therefore, while TiO_2_:SiO_2_ 1:1 and TiO_2_:SiO_2_ 1:3 are totally ineffective on A549, TiO_2_:SiO_2_ 3:1 is able to exert a cytotoxic effect, based on ROS-dependent necrosis, autophagy, and cell cycle arrest.

Although TiO_2_ NPs exposure can induce limited cytotoxicity, they can affect/disassembly actin and tubulin networks with consequent alterations in the cytoskeleton. In addition, proteomic analyses have evidenced that some alterations in proteins related to cytoskeleton disturbances may occur after TiO_2_ NPs exposure [62].

No significant alterations have been observed in the morphology of cells exposed to the three types of silica-modified TiO_2_ nano formulation with hematoxylin and eosin (HE/E) staining. Nevertheless, high magnification images show that the interaction between cells and TiO_2_:SiO_2_ 1:1 and 3:1 are more similar to each other and to TiO_2_ NPs compared to NPs 1:3, as also shown by side scattering analyses. Moreover, further investigations on the actin microfilaments showed that the exposure to TiO_2_:SiO_2_ 1:1, 3:1, and TiO_2_ induced modification of cellular cytoskeleton. In particular, the increased localization of actin protein at the cellular cortex, loss of stress fibers, and increased filopodia could be due to the more evident interaction between cells and NPs.

Cytoskeleton alterations may be related to oxidative stress and inflammation even under low concentrations of NP exposure, as well as direct effects on cytoskeleton components.

The different distribution of NPs within A549 cells is in line with their physicochemical parameters: TiO_2_:SiO_2_ 1:1 and 3:1 have dimensions in the nanometric range (126 and 148 nm, respectively), highly positive surface charge and low polydispersity index are favorable to efficient interaction with cells. The lower positive ζ-potential and the higher PdI of TiO_2_:SiO_2_ 1:3 indicates that these NPs are less stable in water suspension and tend to form big agglomerates, as also proved by the large hydrodynamic size and confirmed by microscopy observation.

Previous works have reported that the silica coating reduces the toxic effects of other antimicrobial NPs, such as zinc oxide (ZnO) and iron oxide (Fe_3_O_4_) nanoparticles [17,18]. Silica prevents zinc and iron toxicity because it improves their stability in the biological fluids, reducing oxidative stress and their overall cytotoxicity and genotoxicity. Moreover, bare NPs release more zinc and iron ions in the intracellular space, with a stronger in situ degradation. In our case, encapsulation is likely not working by preventing ions dissolution from the NP surface, even considering that TiO_2_ is poorly solubilized in an aqueous cellular medium [63]. It is more likely that SiO_2_ encapsulation is able to mask the titania crystal surface, which in turn may produce local ROS once in contact with intracellular molecules.

## 5. Conclusions

In recent studies, SiO_2_-modified TiO_2_ NPs have been proposed as photocatalytic and anti-bacterial devices for surface coatings of different materials (e.g., textiles). Silica coating increases the photocatalytic activity of titania NPs and at the same time reduces the potential toxic effects induced by TiO_2_. The current application of photoactive TiO_2_ nanoparticles in many industrial fields raises the crucial issue of their safety both for workers, in the production sites, and end-users. In this scenario, our study not only confirms the relevance of silica coating as a powerful strategy to prevent the adverse impact of TiO_2_ NPs but also provides an exact indication on the most effective SiO_2_ vs. TiO_2_ ratio into the nanocomposite. Higher protection is obtained in recent studies. SiO_2_-modified TiO_2_ NPs have been proposed as photocatalytic and anti-bacterial devices for surface coatings of different materials (e.g., textiles). Silica coating increases the photocatalytic activity of titania NPs and at the same time, reduces the potential toxic effects induced by TiO_2_. The major toxic effects induced by TiO_2_ are triggered by oxidative stress, which leads to cellular necrosis and DNA damage. The size and physical properties of NPs are partly responsible for the NPs toxicity, with particles with smaller size and higher stability are more interactive with pulmonary cells. The encapsulation of TiO_2_ NPs in a SiO_2_ matrix with the higher content of SiO_2_ vs. TiO_2_ decreases the cytotoxic effects of TiO_2_.

The major toxic effects induced by TiO_2_ are triggered by oxidative stress, which leads to cellular necrosis and DNA damage. The size and physical properties of NPs are partly responsible for the NPs toxicity, with particles with smaller size and higher stability being more interactive with pulmonary cells. The chemical composition of NPs and the ratio among silica and titania plays a crucial role in the induction of the observed biological effects posing great attention in the design of these nanomaterials for their safe application in several industrial and research fields.

## Figures and Tables

**Table 1:** Colloidal properties of NPs. Hydrodynamic diameter (Z-average), polydispersity index (PdI) and zeta potential (ζ-potential) of TiO_2_:SiO_2_ nanoparticles (NPs) in water.

Sample	pH	d_DLS_ (nm)	PdI	Zeta-pot_ELS_ (mV)
SiO_2_	9.0	20.4 ± 0.3	0.2	−42.9 ± 1.1
TiO_2_	1.5	53.0 ± 0.9	0.2	+38.3 ± 1.8
TiO_2_:SiO_2_ 1:1	5.0	125.7 ± 1.26	0.4	+40.5 ± 0.3
TiO_2_:SiO_2_ 1:3	5.0	1490.3 ± 829	0.8	+7.08 ± 0.4
TiO_2_:SiO_2_ 3:1	5.0	147.8 ± 2.3	0.3	+46.1 ± 0.3

## Supplementary Materials

The following are available online at https://www.mdpi.com/2079-4991/9/7/1041/s1, Figure S1: TEM images of a) TiO_2_ and b) SiO_2_ nanoparticles. Figure S2: Cell viability was assessed by MTT test after 24h of exposure to increasing doses of TiO_2_ (dark grey bars) and SiO_2_ (grey bars) NPs. Figure S3: Hoechst/PI test and Annexin V/PI assay for the evaluation of necrotic and apoptotic cells. Figure S4: Oxidative stress was evaluating by detecting ROS in A549 after 180 min of exposure to TiO_2_ and SiO_2_ NPs (75 µg/mL) and positive control H_2_O_2_ (100 µM) by using the fluorescent probe DCFDA. Figure S5: Autophagy was investigated through the analysis of the expression of LC3B II protein by cytofluorimeter. Figure S6: Morphology of cells after exposure to 75 µg /mL of TiO_2_. Figure S7: Morphology of cells after exposure to 75 µg/mL of TiO_2_. Figure S8: Cells and NPs interactions after exposure for 24h to TiO_2_ NPs.
