# Supplementary material for: In Vitro Toxicity of TiO2:SiO2 Nanocomposites with Different Photocatalytic Properties
Source: Nanomaterials (Basel). 2019 Jul 21;9(7):1041. doi: 10.3390/nano9071041 (PMC6669742; doi:10.3390/nano9071041)
Supplement: Supplementary file 1 [file nanomaterials-09-01041-s001.pdf]

Supplementary Materials

In vitro toxicity of TiO_2:SiO_2 nanocomposites with different photocatalytic properties

Rossella Bengalli[1], Simona Ortelli[2], Magda Blosi[2], Anna Costa[2], Paride Mantecca[1], Luisa Fiandra[1*]

[1] POLARIS Research Centre, Dept. Of Earth and Environmental Sciences, University of Milano – Bicocca, Piazza della Scienza 1, 20126, Milano, Italy; rossella.bengalli@unimib.it; paride.mantecca@unimib.it; luisa.fiandra@unimib.it
[2] Institute of Science and Technology for Ceramics (CNR-ISTEC), National Research Council of Italy, Via Granarolo 64, 48018 Faenza, RA, Italy; simona.ortelli@istec.cnr.it; magda.blosi@istec.cnr.it; anna.costa@istec.cnr.it.
[*] Correspondence: luisa.fiandra@unimib.it; Tel.: +390264483406

S1. Characterization of TiO_2 and SiO_2 nanoparticles.

Figure S1a shows a very fine primary structure of the TiO_2 sample with diameter of the primary NPs was about 5 nm, corresponding to the crystal lattice. As reported by DLS data it is confirmed that the NPs were organized in the form of aggregates. The structure of the silica nanosol is monodispersed with spherical primary particles with a mean diameter of around 20 nm (Figure S1b). It also revealed the presence of amorphous SiO_2 particles.

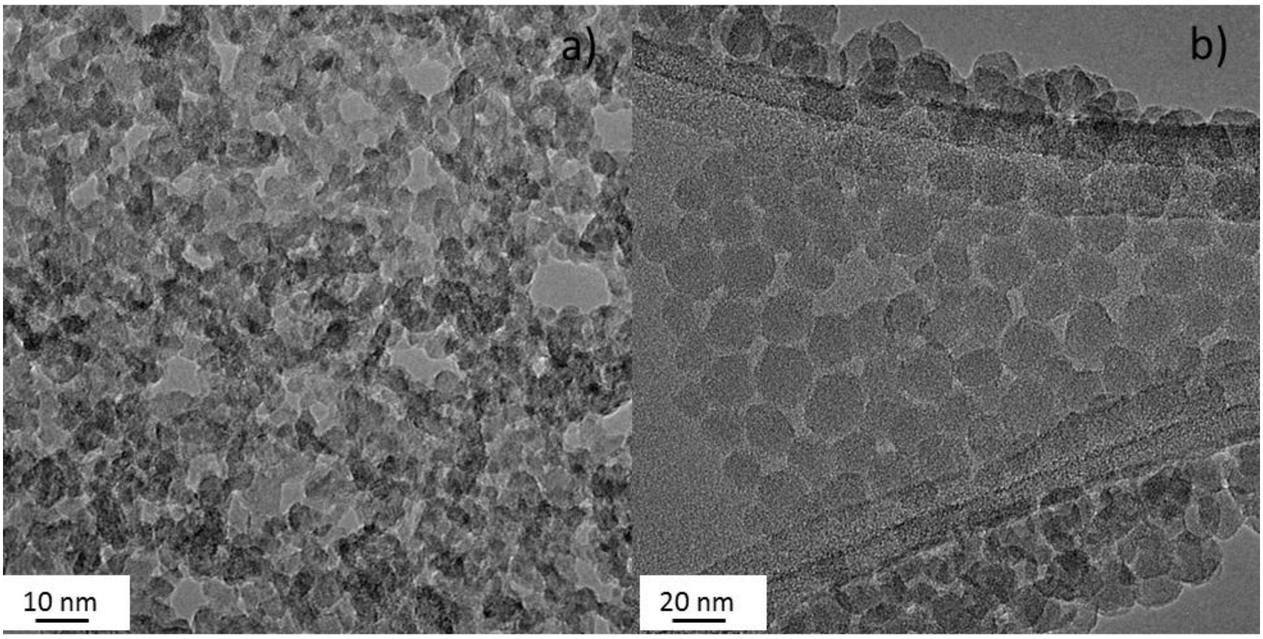

Figure S1. TEM images of a) TiO_2 and b) SiO_2 nanoparticles.

S2. Cytotoxicity on A549 cells of TiO2 and SiO2 nanoparticles

Data of TiO_2 and SiO_2 NPs cytotoxicity on A549 cells were assessed after 24h of exposure. Results show that TiO_2 NPs induced cell death at the highest dose (750 μg/ml), while SiO_2 NPs results toxic even at lower concentration of exposure (Figure S2).
Cell viability data (MTT test) evidenced that SiO_2 is highly toxic compared to TiO_2 NPs, nevertheless it should be taken into account that SiO_2 NPs have a smaller size respect to TiO2 (20 nm *vs* 53 nm) and that in this work we are considering a nanomaterials that has different properties compared to their single constituents. Since data evidenced that TiO_2 and TiO_2:SiO_2 nanoparticles have a similar behavior, as also shown by TEM images,

all the biological endpoints were investigated also after the exposure to the single TiO_2 NPs at the dose of 75 μg/ml. Data from H/PI staining (Figure S3a) show that TiO_2 induce necrosis.

The level of apoptosis and necrosis induced by the different TiO_2:SiO_2 nanocomposite was also evaluated through cytofluorimetric analysis with the Annexin V/PI test (Molecular Probes, Life Technologies, Monza, Italy) (Figure S3b and c). Data showed that TiO_2:SiO_2 3:1 and TiO_2 alone induced an increased expression of Annexin V+/PI + cells, as indication of necrotic cells. A slight increase of apoptosis was also appreciated with this method.

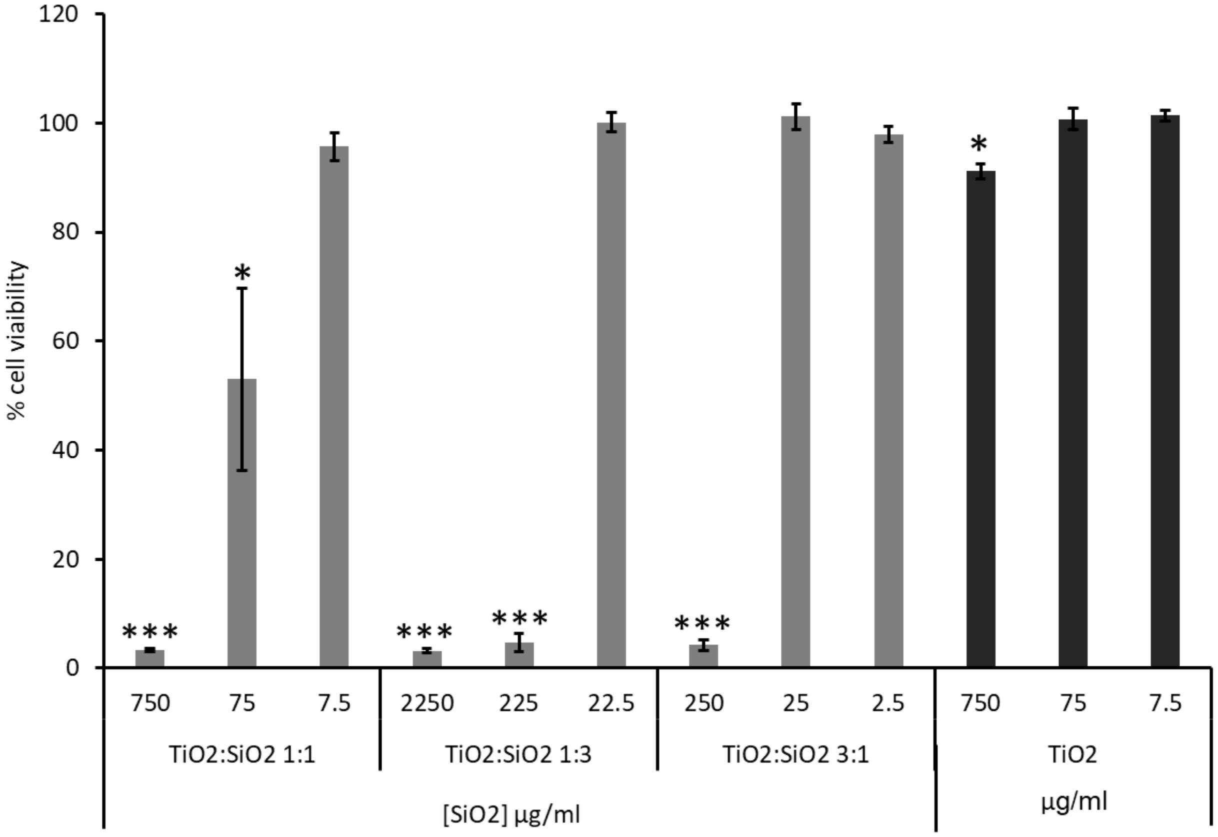

Figure S2. Cell viability was assessed by MTT test after 24h of exposure to increasing doses of TiO_2 (dark grey bars) and SiO_2 (grey bars) NPs. A549 were exposed to SiO_2 doses equivalent to the ones which were present in the different TiO_2:SiO_2 NPs having different TiO_2/SiO_2 ratio. Data show the mean ± SE of at least three independent experiments. *Statistically significant respect to control, ***$p < 0.001$, *$p<0.05$. One-Way ANOVA + Bonferroni's post hoc test.

a)

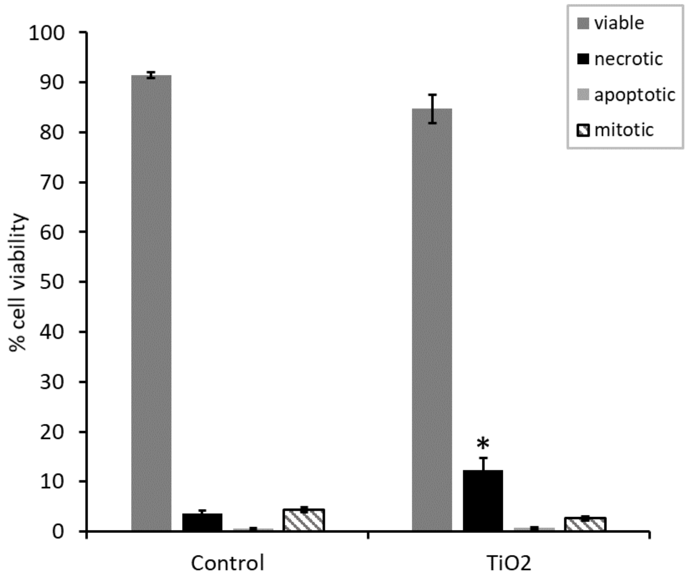
viable
necrotic
apoptotic
mitotic
100
90
80
70
60
50
40
30
20
10
0
% cell viability
*
Control
TiO2

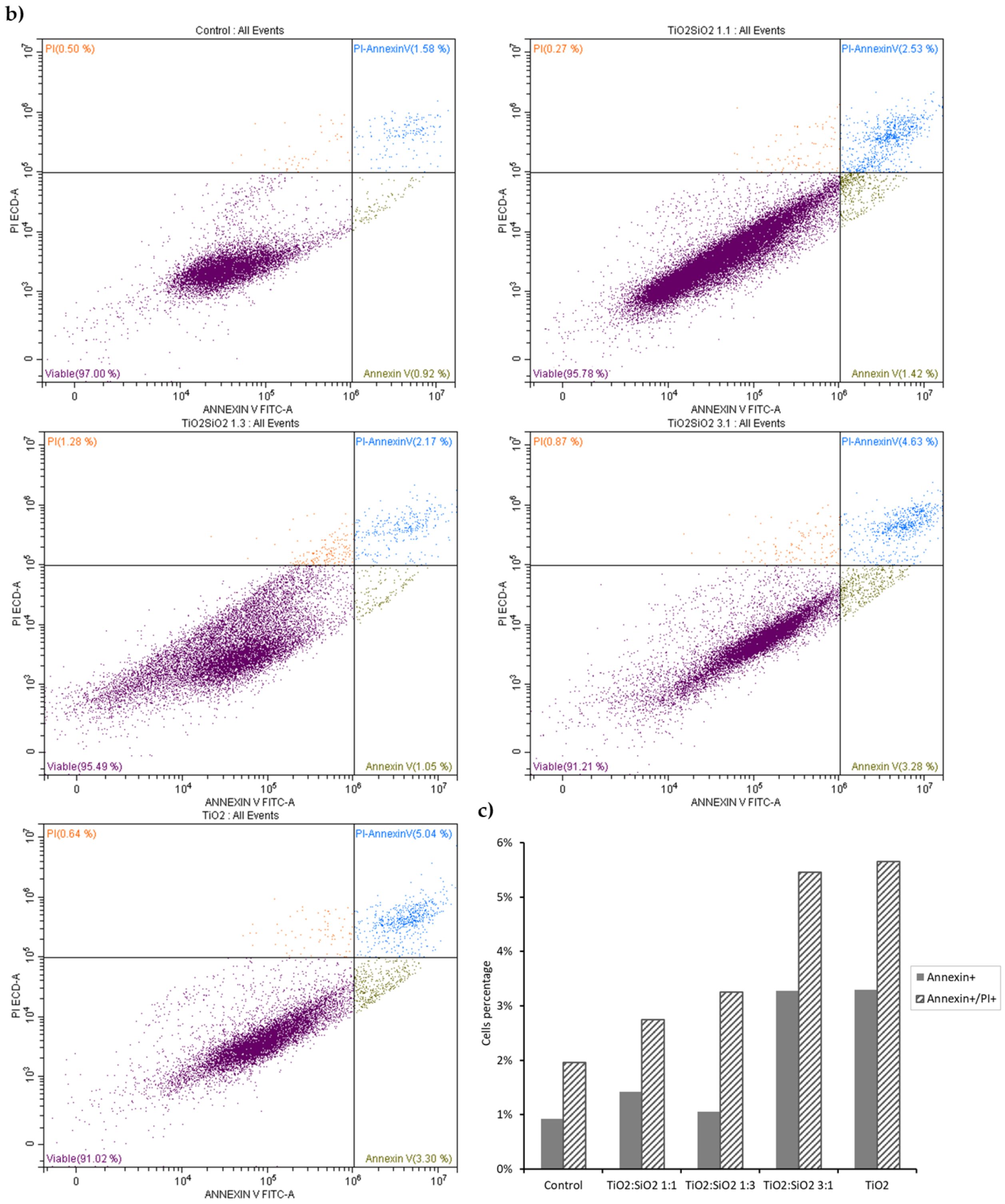

Figure S3. Hoechst/PI test and Annexin V/PI assay for the evaluation of necrotic and apoptotic cells. a) H/PI staining of A549 treated for 24 h with different TiO_2 (75 µg/mL). The histograms represent the percentage of viable (grey bars), necrotic (black bars), apoptotic (light grey bars) and mitotic (dashed bars) cells. Data show the mean ± SE of at least three independent experiments. *Statistically significant respect to control according t-test + Bonferroni's post hoc test; $p < 0.05$. b) Dot plots representing the percentage of positive cells for each staining. PI+/Annexin V+: late apoptotic/necrotic cells, Annexin +: early apoptotic cells, PI-/Annexin V-: viable cells. c) Percentage of necrotic (Annexin+/PI+) or apoptotic (Annexin+) cells after 24 h of exposure to 75 µg/mL of different TiO_2:SiO_2 nanocomposites. Annexin V/PI staining was performed according to the manufacturer's

instructions (Invitrogen/Molecular Probes). Data show the mean percentage of a representative experiment (n=2).

S3. Oxidative stress and autophagy

Oxidative stress was evaluated through the analysis of ROS formation, while LC3B II expression was investigated as marker of autophagy. Data show that TiO_2 NPs induce both ROS (Figure S4) and autophagy (Figure S5), while SiO_2 NPs induce ROS.

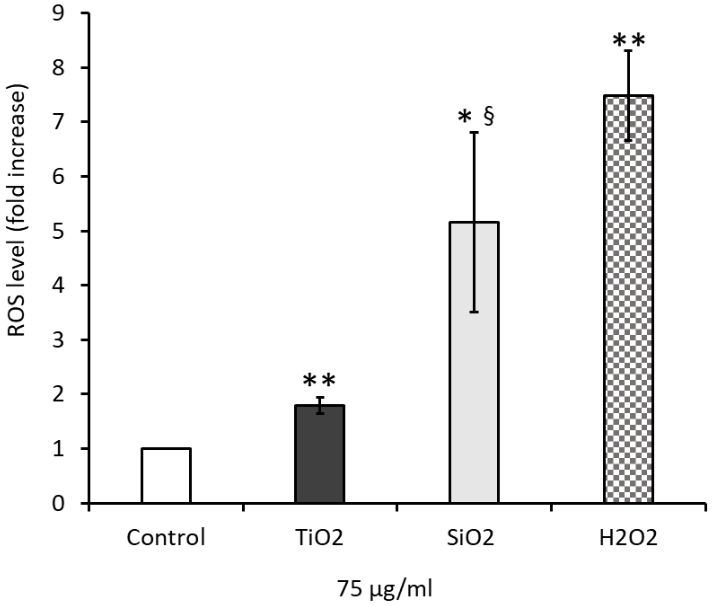

Figure S4. Oxidative stress was evaluating by detecting ROS in A549 after 180 min of exposure to TiO_2 and SiO_2 NPs (75 µg/mL) and positive control H_2O_2 (100 µM) by using the fluorescent probe DCFDA. The histograms represent the mean ± SE of at least three independent experiments. *Statistically significant respect to control; §Statistically different from sample TiO_2; t-test; **$p < 0.001$; *$p < 0.05$.

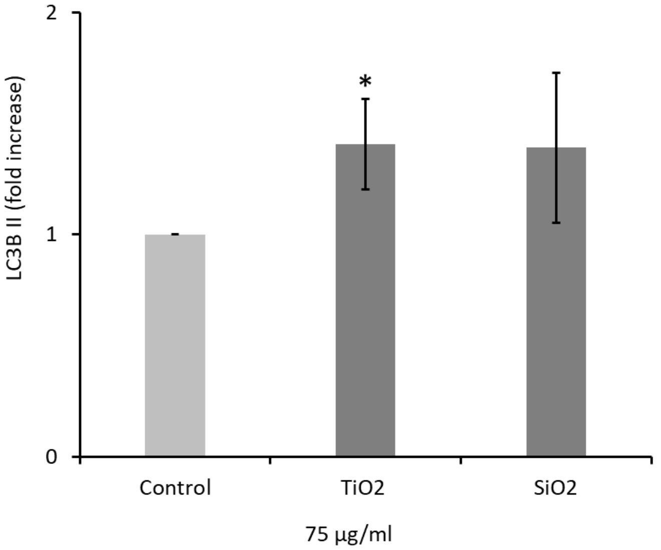

Figure S5. Autophagy was investigated through the analysis of the expression of LC3B II protein by cytofluorimeter. Cells were stained with LC3B II antibody after 24 h exposure to TiO_2 and SiO_2 NPs (75 µg/mL). The histograms represent the fold change of LC3B II expression over the control and they are the mean ± SE of at least three independent experiments. *Statistically significant respect to control; t-test $p < 0.05$

S4. NPs interaction and morphological changes

Data from HE/E (Figure S6) and rhodamine phalloidin (Figure S7) staining show the interaction between A549 cells and TiO_2 NPs. The increased interaction/uptake of TiO_2 NPs is also confirmed by Side Scatter analysis (Figure S8).

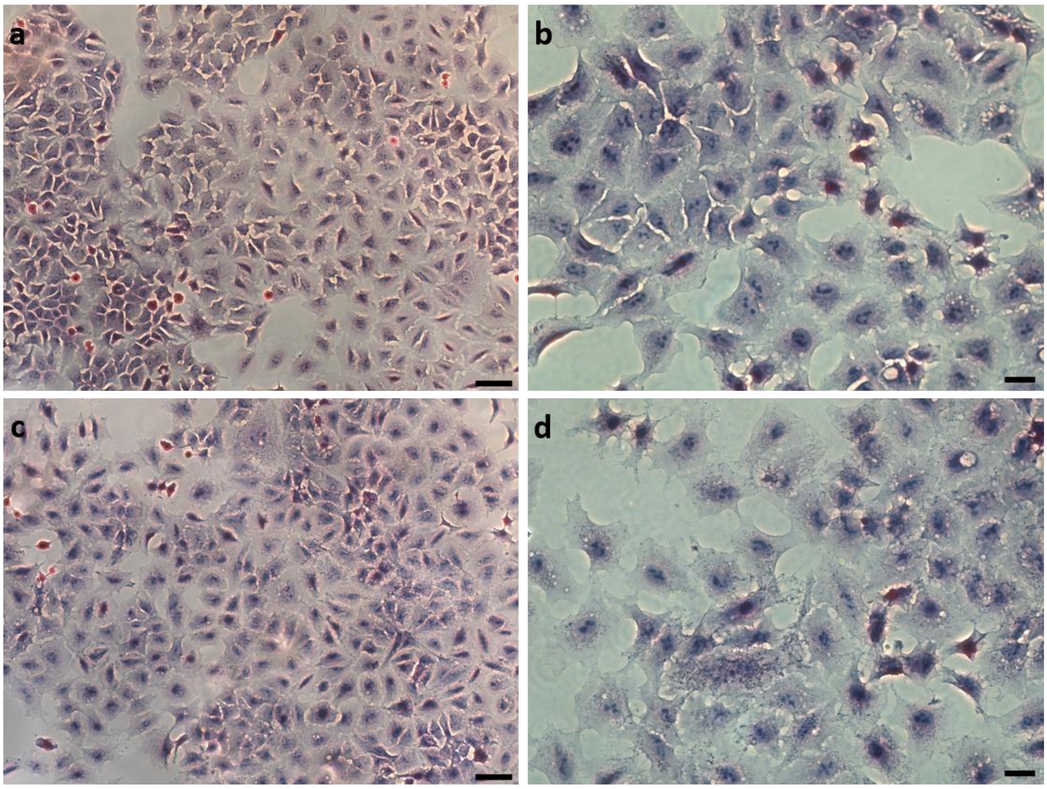

Figure S6. Morphology of cells after exposure to 75 µg /mL of TiO (c, d). Control cells are shown in Figure a and b. Cell were fixed and stained with Haematoxylin/Eosin. Scale bars: 50 µm (a, c); 20 µm (b, d). HM: high magnification of b and d. Black arrows: nanoparticles interacting with cells.

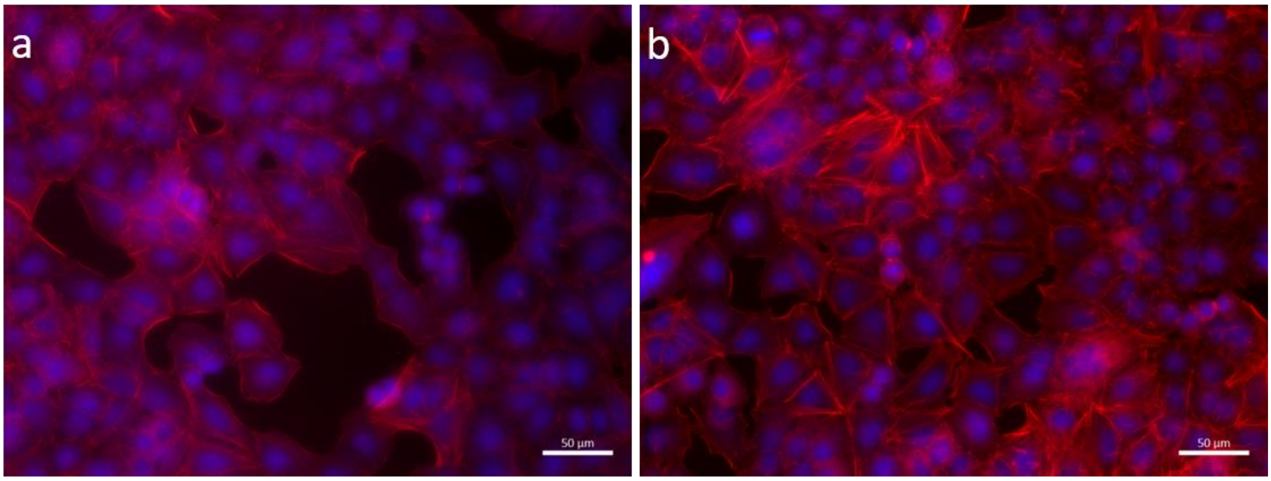

Figure S7. Morphology of cells after exposure to 75 µg/mL of TiO_2 (b). Control cells are shown in Figure a. Cell were fixed and stained with DAPI (blue) and Rhodamine Phalloidin (red). Scale bars: 50 µm.

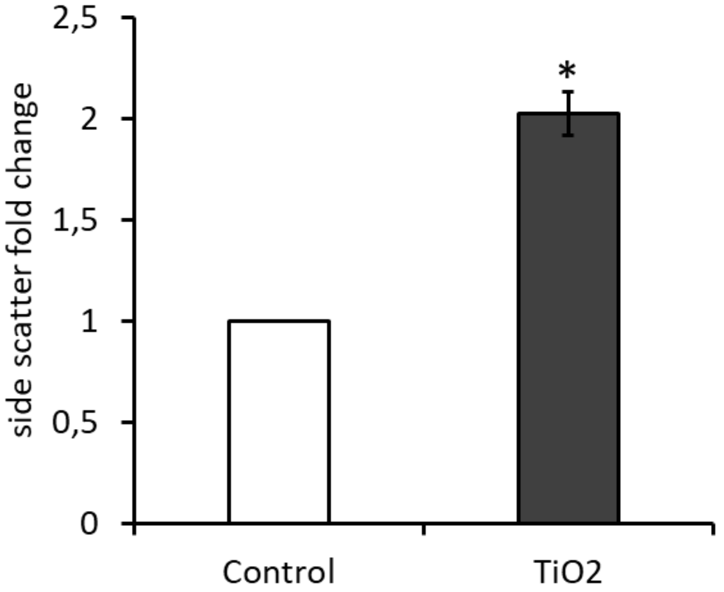

Figure S8. Cells and NPs interactions after exposure for 24h to TiO_2 NPs. The histograms show the fold change of side scatter (SSC) and data represents the mean ± SE of at least three independent experiments. *Statistically significant respect to control sample; unpaired t-test; $p < 0.05$.
